# Effects of Microplastics, Fertilization and Pesticides on Alien and Native Plants

**DOI:** 10.3390/plants13212947

**Published:** 2024-10-22

**Authors:** Xiong Shi, Guilin Yang, Yulong Zheng

**Affiliations:** 1 CAS Key Laboratory of Tropical Forest Ecology, Xishuangbanna Tropical Botanical Garden, Chinese Academy of Sciences, Menglun, Mengla 666303, China; shixiongsnnu@gmail.com; 2 University of Chinese Academy of Sciences, Beijing 100049, China; 3 College of Biology and Chemistry, Puer University, Puer 665000, China; 15287676157@139.com

**Keywords:** nutrient, plastic mulch, specific leaf area, biomass allocation, plasticity

## Abstract

Plastic mulches, fertilizers and pesticides have been extensively employed in agriculture to increase crop yields, though it has also led to the inadvertent accumulation of them over time. These accumulations have the potential to disrupt the soil ecological process and subsequently impact the plant community composition. Alien plants always benefit from environmental variability, thus whether the accumulation of fertilizer, plastic, and pesticide in soil promotes the dominance of alien plants in an invaded community. Here, five aliens and co-occurring natives were selected as study materials, and a full factorial experiment was conducted to answer this question. Our study found that microplastics promote the biomass production of native plants at higher nutrient availability while having marginal influence on growth of alien plants. Alien plants exhibited a lower root mass fraction (RMF) with increased nutrient availability and a higher specific leaf area (SLA) in response to the addition of nutrients and microplastics. Pesticide residues in the soil also significantly decreased the root mass fraction of three species, but there was no significant difference between the effects on alien and native species. Overall, our results revealed that alien species adjusted their functional traits more quickly, but native species gained more growth advantages in response to fertilization and microplastics.

## 1. Introduction

To meet the exponential food demand with ongoing population growth, several agronomic practices have been simultaneously employed to enhance crop yield, including the use of plastic mulches, fertilizers and pesticides [1,2,3,4,5]. However, long-term agricultural practices as described above resulted in the inadvertent accumulation of nutrients, pesticides and microplastics in soil [6,7,8]. These residues have the potential to alter soil ecological processes, such as soil (physical and chemical properties) properties, soil nutrient cycle and biota activities [9,10], which might influence plant growth [11,12,13,14,15,16]. In the meanwhile, cropland abandonment is still increasing in many regions and countries around the world [17]. Hence, the accumulation of microplastics, fertilizers and pesticides might have profound impacts on the subsequent vegetation community composition; it is urgent to address this important question to explore the potential mechanisms underlying the process.

Numerous studies have revealed that fertilization plays a significant role in vegetation composition and individual plant growth [11,12,13]. However, few studies have tested how other agricultural practices (including microplastics and pesticides) influence plants [14,15,16]. More importantly, the application of plastic mulch, pesticide and fertilizer typically occurred together rather than individually, and there might be interactive effects between fertilization and microplastics or pesticides on plant performance. For instance, a recent study found that phosphorous addition could reduce the adverse effect of microplastics on rice growth [18], and Tripathi et al. [19] suggested that both excessive fertilization and pesticide could lead to soil degradation, which might have a strong synergistic negative effect to the latter vegetation composition. However, there is still a knowledge gap regarding how these residues collectively influence vegetation composition.

Generally, agricultural practices will directly influence plant community at highly disturbed sites, such as abandoned agricultural land [20] and field margin [21]. Meanwhile, all these districts are also frequently distributed habitats of alien plants [22,23]. Indeed, previous studies have revealed the positive effect of environment variability on alien plant invasion [24], and some studies have also suggested that alien plants could benefit from numerous environmental change scenarios (including increasing CO_2_, warming and artificial light at night) [25,26,27]. Although anthropogenic activities were considered as a key driver of plant invasion [28], whether the accumulation of these three residues will promote alien plant invasion remains uncertain.

Numerous studies have suggested that alien plants could benefit from environmental change, which is attributed to its rapid adjustment for functional traits [29,30]. For instance, a common garden experiment revealed that invasive grass (*Imperata cylindrica*) exhibited greater plasticity in biomass allocation than six co-occurring natives under shading [31]. Another study also suggested that *Chromolaena odorata* will increase specific leaf area (SLA) more than three co-occurring natives in response to warming [32]. Generally, the rapid adjustment for the above traits could help alien plants tolerate stressful environments or promote their resource utilization efficiency in a favorable environment [33]. Henceforth, a greater plasticity of biomass allocation and SLA might contribute to the invasion success of alien plants under ongoing environment condition changes.

Yunnan province, located in Southeast Asia, is also simultaneously undergoing the rapid accumulation of fertilizer, microplastic and pesticide residues [6,8,34] and severe plant invasion [35]. Five alien plants and five co-occurring native plants in Yunnan were selected as our study materials. A common garden experiment, containing individual and interactive treatments of fertilization, microplastic and pesticide addition, was conducted to test the effects of microplastics, fertilization and pesticides on the performance of alien and native plants. We try to answer the following questions: (1) How does the accumulation of the three residues influence alien and native plants’ growth? (2) Do alien plants have a greater performance in biomass allocation and specific leaf area during this process?

## 2. Materials and Methods

### 2.1. Study Site and Species

A full factorial experiment was designed to test the effects of fertilization, microplastics and pesticides on alien plant invasion. This study was conducted in the greenhouse at Xishuangbanna Tropical Botanical Garden (21°56′ N, 101°15′ E), Southwest China. In this district, the average annual temperature is 21.7 °C, with the hottest month being July (mean temperature: 25.3 °C) and the coolest month being January (mean temperature: 15.6 °C). This area experiences a mean precipitation of 1557 mm, with a dry period extending from November to April. For the greenhouse, the maximum and minimum temperature are 36 °C and 13 °C, respectively, with a humidity of approximately 70% and light intensity of about 800 μmol m^2^ s^−1^. To increase the generality of our results, we selected a total of 10 terrestrial plants (5 alien species: *Ageratina adenophora* (Spreng.) R.M.King & H. Rob., *Bidens pilosa* L., *Chromolaena odorata* (L.) R.M.King & H.Rob., *Phytolacca americana* L. and *Tithonia diversifolia* (Hemsl.) A.Gray; and 5 native species: *Coix lacryma-jobi* L., *Cyanthillium cinereum* (L.) H. Rob., *Laggera crispata* (Vahl) Hepper & J. R. I. Wood, *Puhuaea sequax* (Wall.) H.Ohashi & K.Ohashi and *Senecio scandens* Buch.-Ham. ex D.Don) co-occurring in Yunnan. To classify the species as alien or native according to the POWO database (https://powo.science.kew.org/ (accessed on 9 July 2024)), species details are listed as Table S1.

### 2.2. Experimental Design

**Figure 1:**
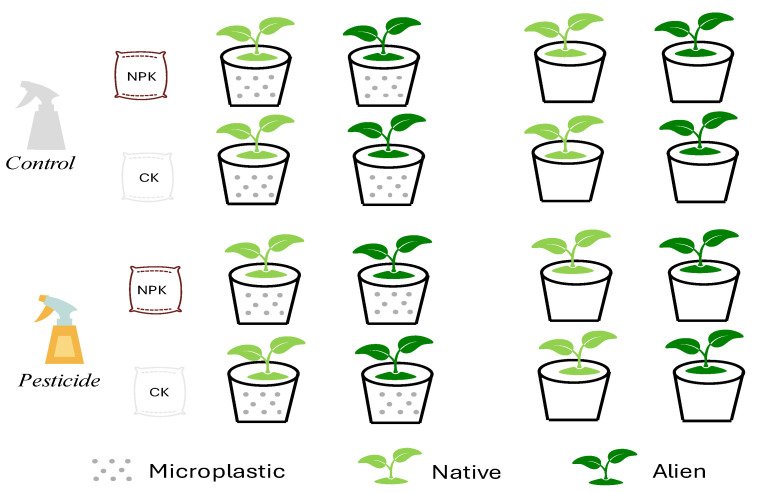
Graphical illustration of the experimental design. The common garden experiment consisted of eight soil types (2^3[fertilizer, plastic, pesticide]^), and there were 400 pots in total with 5 replicates of each soil type encompassing ten species (5 aliens, 5 natives).

Given the challenges in controlling variables during field soil collection and the potential interference from other factors, we simulated the effects by adding three types of residues. Soil conditioning phase: we mixed field soil and sand together at a 2:1 ratio. Subsequently, we applied microplastics, fertilizers and pesticides individually and interactively, resulting in eight unique soil types (shown in Figure 1). Polyethylene (PE) is a common microplastic found in farmland soil [36], and thus we used PE (150 µm, Hongxing Polymer Company, Dongguan, China) as a microplastic treatment (2% of the soil fresh weight) in this study [15]. Moreover, we also purchased the popular fertilizer (Lion Horse Nitrophoska 15-15-15, EuroChem, Antwerp, Germany) and pesticide (indoxacarb, Zhongbaolvnong S&T Group Company, Langfang, China) from a local shop, and then 1.5 g of fertilizer and 0.01 mL of pesticide were incorporated per pot, following the recommended product dosages.

Transplanting phase: We put all seeds into plastic trays (42 cm × 42 cm × 4 cm) filled with potting soil (Pindstrup, Fabriksvej, Denmark). On 29 July 2022 (one month after), we selected similar-sized seedlings (identical height for the same species) and then transplanted them into one individual per pot (height 17.5 cm, diameter 16 cm). If any seedlings died within the first week, we would replace them in time. To mitigate position effects, all pots were randomly arranged and repositioned every twenty days. We watered them every two days. There were 400 pots in total (8 soil × 10 species × 5 replicates).

### 2.3. Measurement

On 9 November 2022, we initially measured the plant height (from the base of the stem to the top of the canopy) and collected well-developed leaves for each plant. Subsequently, we measured leaf area with a leaf area meter (Li-3100; Li-Cor Inc., Lincoln, NB, USA) and its biomass parts after being dried to constant mass at 80 °C for 48 h (DHG-9620A, Yiheng, Shanghai, China); the specific leaf area (SLA) was calculated by dividing leaf area by the dry mass of leaves. We then carefully collected the root and above parts, and all plant parts were weighted with similar methods as mentioned above. We also calculated the root biomass fraction (RMF) as root biomass/total biomass.

### 2.4. Statistical Analyses

All analysis was performed in R version 4.2.1 [37]. To test the individual and interactive effects of three residues on alien and native plants, six linear mixed models were constructed to analyze the total biomass, root biomass, leaf biomass, stem biomass, height, RMF and SLA of the plants with the *nlme* package [38]. For all models, we include plant origin, fertilization treatment, microplastic treatment, pesticide treatment and their two- and three-way interactions as fixed factors (four-way interaction is impossible to interpret clearly; we did not consider it), and plant species were regarded as random factors. To improve the normality of the residuals, we applied a log transformation to the stem biomass and utilized a square root transformation for the other response variables. To improve the homoscedasticity of the residuals, the species was allowed to have different variances by using the *varComb* and *varIdent* functions [39].

To test the individual response for each species, we performed an ANOVA test for 7 traits (total biomass, root biomass, leaf biomass, stem biomass, height, RMF and SLA) across ten species, and we just show the effects of individual factor, and the interaction among the three factors was treated as fixed factor (the three-way interaction was omitted because it is too complex to explain). To improve the normality of model residuals, we performed data transformation for some models (details can be found in Table S2).

## 3. Results

### 3.1. Effects of Fertilizer on Alien and Native Plants

**Figure 2:**
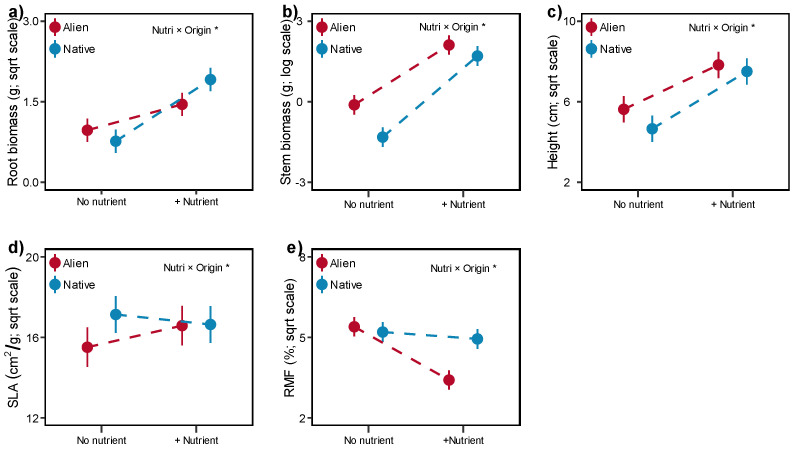
Effects of nutrient (Nutri) on the root biomass (**a**), stem biomass (**b**), height (**c**), SLA (specific leaf area, (**d**)) and RMF (root mass fraction, (**e**)) of alien and native plants. Error bars indicate standard error. Significant effects (asterisk) are indicated in the right corner (details in Table 1).

Fertilization significantly promoted biomass production and height in both alien and native plants (Table 1, Figures S1–S6). However, native plants benefit more in root, stem and height than alien plants (Table 1, Figure 2a–c). In contrast, alien plants showed an increase in SLA, while native plants showed a decrease, and a greater reduction in RMF of alien plants was also observed with nutrient addition (Table 1, Figure 2d,e). Furthermore, three of five alien species (*B. pilosa*; *C. odorata*; *P. americana*) increased their SLA, while none of the native plants have a positive response to fertilization (Figure S7). Moreover, both five alien species reduce their root mass fraction after fertilization, with a similar pattern being found in three native species (*C. cinereum*, *P. sequax*, *S. scandens*, Figure S8).

### 3.2. Effects of Microplastics on Alien and Native Plants

**Figure 3:**
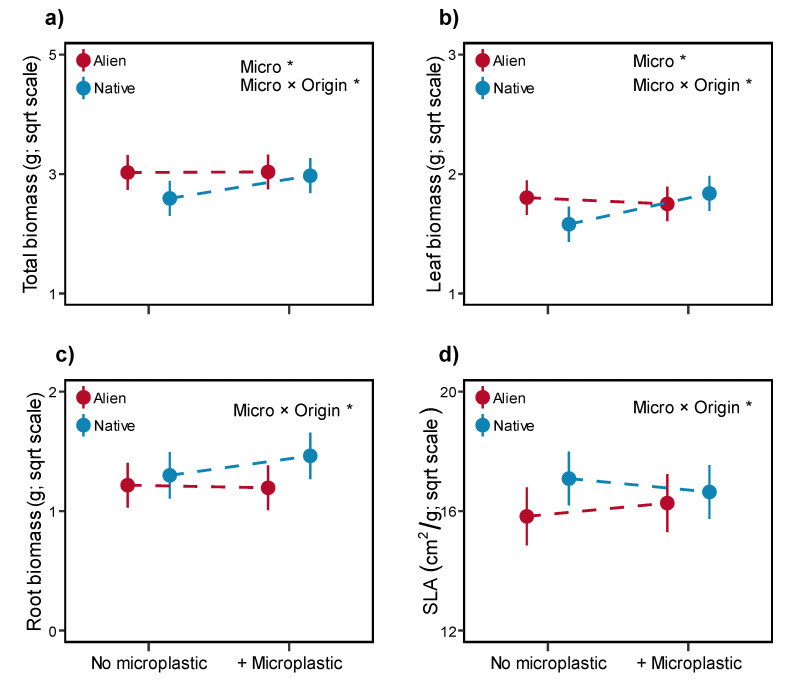
Effects of microplastics (micro) on total biomass (**a**), leaf biomass (**b**), root biomass (**c**) and SLA (specific leaf area, (**d**)) of alien and native plants. Error bars indicate standard error. Significant (asterisk) effects are indicated in the right corner (details in Table 1).

The presence of microplastics promotes the total and leaf biomass accumulation, but native species responded more positively to microplastics in total and leaf biomass, whereas aliens exhibited a slight decrease for the above two traits (Table 1, Figure 3a,b). We also observed significant different responses on root biomass (alien: −3.6%, native: +26.65%) and SLA (alien: −3.6%, native: +10.3%, Table 1, Figure 3c,d). Although there was a positive response to microplastic addition for alien *P. americana* in total biomass, but *B. pilosa* and *C. odorata* have a negative response to microplastics in biomass production (*B. pilosa*: total and leaf biomass, *C. odorata*: total, leaf and root biomass); no native species exhibit a negative response to microplastics, and four native species (*C. lacry-jobi*, *C. cinereum*, *L. crispate*, *P. sequax)* exhibit a positive response in the above three biomass traits (*C. lacry-jobi:* leaf; *C. cinereum:* total, leaf; *L. crispate:* total, leaf; *P. sequax:* total, root; Figures S2–S6). For SLA, we could see that microplastic addition significantly reduced the SLA of three native species (*C. lacryma-jobi*, *L. crispata*, *S. scandens*), but alien *C. odorata* respond positively to microplastics (Figure S7).

### 3.3. Effects of Pesticides on Alien and Native Plants

**Figure 4:**
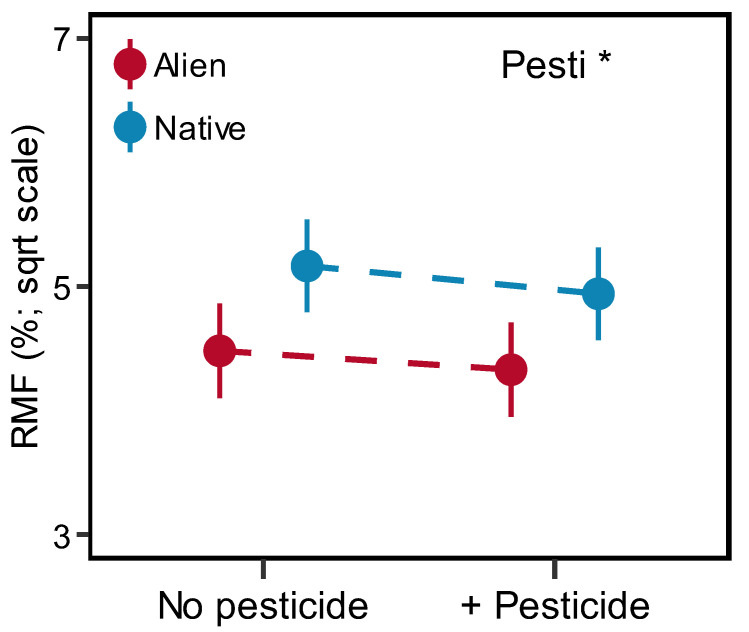
Effects of pesticide (Pesti) on the RMF (root mass fraction) of alien and native plants. Error bars indicate standard error. Significant (asterisk) effects are indicated in the right corner (details in Table 1).

Overall, the addition of pesticides generally reduced the biomass allocation to the roots (Figure 4). Specifically, the RMF of three species (*B. pilosa*, *L. crispate* and *S. scandens*) was reduced after pesticide addition (Figure S8). However, only a few species have a significant response to pesticides for the other six traits (Figures S2–S7).

### 3.4. Interaction Effects of Nutrients and Microplastics on Alien and Native Plants

**Figure 5:**
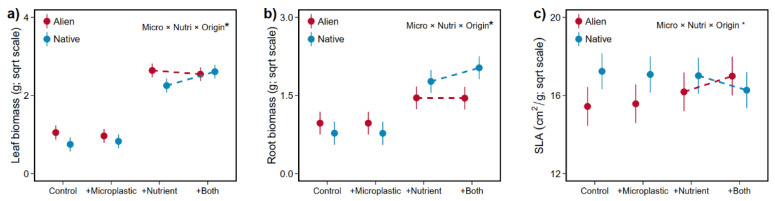
Interaction effects of microplastic (Micro) and nutrient (Nutri) addition on the leaf biomass (**a**), root biomass (**b**) and SLA (**c**) of alien and native plants. Error bars indicate standard error. Significant (asterisk) effects are indicated in the right corner (details in Table 1).

More importantly, our findings revealed that native species responded more positively to microplastics in the leaf and root biomass (+34.2%, +31.7%) with increased nutrient availability, whereas aliens exhibited a slight decrease for the above two traits (−7.0%, −0.7%, Table 1, Figure 5a,b). We also found that alien plants exhibited an increase in SLA (+10.2%), contrasting with a decrease of 8.5% in native plants, both in response to microplastics at higher nutrient availability (Table 1, Figure 5c). Although no significant interaction between microplastic and fertilization was found, microplastic treatment increased the leaf biomass after fertilization, but four alien species (*B. pilosa*, *C. odorata*, *P. americana*, *T. diversifolia*) showed opposite patterns (Figure S3). For root biomass, four native species (*C. lacryma-jobi*; *C. cinereum*; *L. crispate*; *P. sequax*) produced more root biomass with the joint increase in microplastics and nutrients compared with the increase with nutrients alone (Figure S5). However, only two native species showed this pattern (*C. odorata*, *T. diversifolia*, Figure S5). For SLA, both alien species tended to increase, but both native species tended to decrease their SLA after microplastic addition in nutrient-rich environments (Figure S7).

## 4. Discussion

Our study tested how the accumulation of three residues (microplastic, fertilizer and pesticide application) influenced the performance of alien and native species. We found that fertilization had a significantly positive effect on the growth of both plants, with more pronounced positive effects in native species. Furthermore, the positive response of native plants to fertilization was obvious in the presence of microplastics. Overall, these results indicate that microplastic accumulation might promote the growth advantage of native plants in nutrient-rich environments.

Generally, alien plants exhibit a greater root foraging scale and arbuscular mycorrhizal colonization than native plants, enabling them to absorb more nutrient from the surrounding soil [40,41], and numerous studies have also confirmed the positive effect of fertilization on plant invasion [27,42]. However, our present findings revealed a contrasting trend; even though we only applied a single-dose treatment, native plants could benefit more from nutrient addition in root and stem growth (Figure 2). We subsequently found that adding microplastic promoted both plant part growth with increased nutrient availability (Figure 3). Thus, one possible explanation for the discrepancy could be the presence of microplastic.

It has been suggested that microplastics have the potential ability to improve soil structure and water holding capacity [43] or mycorrhizal associations [14]. Microplastics may help roots absorb additional nutrients under higher nutrient conditions by lowering root penetration resistance, whereas their effect may be negligible in control groups with limited nutrient availability. For instance, Liu et al. [44] demonstrated that polyethylene (PE) enhances the absorption of additional nitrogen for wheat (*Triticum aestivum*), and Shi et al. [45] suggested that PE also could help sweet potato (*Ipomoea batatas*) absorb phosphorous and potassium. Thus, we infer that native plants might benefit from the amelioration of microplastic, which may increase the uptake of unused nutrients after fertilization, while alien plants may not derive an additional benefit due to their inherent greater nutrient acquisition strategy. Consistent with our result, a previous study suggested that a lower concentration of EPDM (ethylene propylene diene monomer) will promote the growth of the native plant *Plantago lanceolata* [16]. However, our study does not advocate for the use of microplastics in controlling plant invasion, especially given their risk on human health [46]. Instead, we aim to emphasize that soil improvement—such as improved soil structure, soil moisture and increasing fertility—might promote the competitive advantage of native plants. According to the recent synthesis [47], the effect of microplastics on plant growth is dependent on size and type. Because we only used polyethylene (PE) as the microplastic treatment, additional studies are needed to draw more generalized conclusions.

Moreover, we did not find evidence that pesticide accumulation in the soil influenced plant growth; however, it decreased RMF in both alien and native plants. It could be that with the addition of pesticides, there would be less herbivory, allowing plants to reduce root biomass allocation and increase leaf construction. Another study also suggested that plants would reduce their root allocation when soil fauna were removed in well-watered conditions [48]. Moreover, although the presence of pesticides could alleviate root herbivory, we still did not observe a positive effect on plant growth. One possible explanation for this might be that there was the adverse effect of the pesticide on chlorophyll, protein content and photosynthesis efficiency [49]. However, we did not measure any physiological indices in this study, and it might be beneficial to conduct further experiments to explore the potential mechanism. Additionally, given the wide variety of pesticide available on the market, more pesticide types should be included to draw a more comprehensive conclusion. Nevertheless, our study still found that the accumulation of pesticide in soil might influence the biomass allocation strategy of certain plants.

In addition to functional traits, a global meta-analysis revealed that nitrogen addition will promote terrestrial plants to invest more resources to the aboveground part [50], which was also consistent with our findings (Figure 4). More importantly, our study revealed that aliens reduced more RMF than native plants with increased nutrient availability; Funk [51] also suggested that invasive plants could be more plastic in biomass allocation when treated with fertilization. With unlimited belowground resources, the lower RMF could enable alien plants to allocate more resources to leaf and stem construction, enhancing light capture [52]. Moreover, we found that aliens improved their SLA while natives exhibited a decrease with microplastic addition at higher nutrient availability. Indeed, SLA is also one of the most important functional traits related to light capture and light use efficiency [52,53]. Although we have no direct evidence, we infer that alien plants began to allocate more resources to leaf area construction for light competition after perceiving that soil microplastics ameliorates soil structure, like aeration and porosity [14]. For instance, the presence of polyester increased the volume of pores larger than 30 μm [54], and another review discussed that microplastics could reduce soil bulk density and potentially enhance soil aeration [55]. However, we did not measure any soil indices, further long-term studies are also needed to explore the potential mechanism behind this process.

We did find that alien plants showed quicker adjustment for RMF and SLA in response to fertilization and microplastics, while we did not find alien plants having a better performance than natives in growth (both biomass and height). One possible explanation could be that there was still no strong light competition due to low-density planting (one individual per pot). However, given the intensive light competition in natural communities, alien plants might also benefit from the greater plasticity of the above traits. Secondly, the experiment’s duration was relatively short, lasting only nine weeks, during which the alien species responded quickly but did not yield an immediate return. Moreover, higher phenotypic plasticity is not always positively related with growth due to its cost [56]; instead, it mostly reflects the niche breadth for species coexistence [57].

Finally, although we want to derive a general conclusion via multiple species experiment, we still could not ignore the species-specific effect within group. For instance, the overall effect of microplastic is neutral for alien plants, and we found that *Bidens pilosa* and *Chromolaena odorata* have negative responses to microplastics, while *Phytolacca americana* exhibit a positive response in terms of total biomass (Figure S2). A similar pattern was found in another study, where the authors found that microplastics promoted the reproduction biomass of *Phytolacca americana* more than congeneric alien species [58]. Additionally, although we did not observe a significant main effect of the pesticide on plant growth, we found its significant influence on the height of *Coix lacryma-jobi* (negative), *Cyanthillium cinereum* (negative), *Laggera crispata* (positive) and *Bidens pilosa* (positive) (Figure S6). We also found significant interactions between microplastics and pesticides, as well as between nutrients and pesticides, affecting the growth of certain plants (such as Figure S3g,h). However, studies testing the effect of pesticides and its interaction with fertilization or microplastics on plant performance is relatively rare, so additional evidence is needed to further explore the area. Consequently, these results indicate that species’ specific effects should be considered in biological invasion control and management strategies.

## 5. Conclusions

Overall, our study suggested that the accumulation of microplastics could promote native growth after fertilization, but not for alien plants. Such differences might enhance the growth advantages of native plants in areas where fertilization and plastic mulching occur simultaneously. However, we still found that alien plants exhibited greater adjustment for several functional traits with the input of nutrients and microplastics, suggesting their rapid response for environment changes. Additionally, the pesticide residue influenced plant functional trait but not plant growth, which might also alter the biomass allocation of certain plants.

## Figures and Tables

**Table 1:** Results of linear mixed models testing the effects of the addition of microplastics, nutrients and pesticides, origin, and their interactions on the total biomass, leaf, stem, root biomass and SLA of plants.

	df	Total Biomass		Leaf		Root		Stem		Height		SLA		RMF	
		χ^2^	*p*	χ^2^	*p*	χ^2^	*p*	χ^2^	*p*	χ^2^	*p*	χ^2^	*p*	χ^2^	*p*
Microplastic (M)	1	5.813	**0.016**	4.497	**0.034**	2.233	0.135	2.695	0.101	0.039	0.843	0.281	0.596	0.084	0.772
Nutrient (N)	1	2442.857	**<0.001**	2861.558	**<0.001**	589.002	**<0.001**	1985.259	**<0.001**	655.092	**<0.001**	2.104	0.147	466.749	**<0.001**
Pesticide (P)	1	0.968	0.325	0.322	0.570	0.244	0.621	0.508	0.476	0.473	0.492	3.418	0.064	6.538	**0.011**
Origin (O)	1	0.622	0.430	0.584	0.445	0.245	0.620	2.348	0.125	0.485	0.486	0.693	0.405	2.050	0.152
M × N	1	7.103	**0.008**	4.844	**0.028**	1.868	0.172	0.045	0.831	0.518	0.472	0.015	0.904	0.254	0.614
M × P	1	0.046	0.831	0.752	0.386	0.024	0.877	0.027	0.868	0.750	0.386	0.007	0.932	0.262	0.609
M × O	1	8.916	**0.003**	26.858	**<0.001**	4.926	**0.026**	2.112	0.146	0.798	0.372	13.157	**<0.001**	3.124	0.077
N × P	1	0.266	0.606	0.110	0.740	0.054	0.817	0.211	0.646	0.025	0.875	0.042	0.838	2.208	0.137
N × O	1	0.073	0.787	0.697	0.404	111.069	**<0.001**	45.667	**<0.001**	8.903	**0.003**	38.463	**<0.001**	198.947	**<0.001**
P × O	1	1.345	0.246	1.939	0.164	0.065	0.799	0.484	0.487	0.863	0.353	0.263	0.608	1.308	0.253
M × N × P	1	0.097	0.755	0.154	0.695	0.542	0.461	0.538	0.463	0.028	0.868	0.298	0.585	0.479	0.489
M × N × O	1	3.797	0.051	5.612	**0.018**	4.990	**0.025**	0.238	0.626	3.088	0.079	5.769	**0.016**	2.022	0.155
M × P × O	1	1.184	0.277	0.639	0.424	1.803	0.179	2.501	0.114	0.006	0.940	0.000	0.999	0.825	0.364
N × P × O	1	0.014	0.907	0.344	0.557	0.587	0.444	0.000	0.993	0.053	0.818	0.153	0.696	0.000	0.986
Random effects		SD		SD		SD		SD		SD		SD		SD	
Family		<0.001		<0.001		<0.001		<0.001		0.001		1.171		<0.001	
Species		0.801		0.385		0.478		0.802		1.440		1.369		0.780	
Residuals		0.515		0.279		0.275		0.478		1.169		0.943		0.456	

Significant effects (*p* < 0.05) are marked in bold.

## Data Availability

The data are all present in the figures.

## Supplementary Materials

The following supporting information can be downloaded at: https://www.mdpi.com/article/10.3390/plants13212947/s1, Table S1: Background information of ten species; Table S2: Details on data transformations in the seven-traits models across the ten species. Figure S1: Effects of nutrient (Nutri) on total biomass (a), leaf biomass (b), root biomass (c), stem biomass (d) and height (e) of plants; Figure S2: Effects of microplastics, nutrients, pesticides and their interactions on the total biomass of ten species; Figure S3: Effects of microplastics, nutrients, pesticides and their interactions on the leaf biomass of ten species; Figure S4: Effects of microplastics, nutrients, pesticides and their interactions on the stem biomass of ten species; Figure S5: Effects of microplastics, nutrients, pesticides and their interactions on the root biomass of ten species; Figure S6: Effects of microplastics, nutrients, pesticides and their interactions on the height of ten species; Figure S7: Effects of microplastics, nutrients, pesticides and their interactions on the SLA (specific leaf area) of ten species; Figure S8: Effects of microplastics, nutrients, pesticides and their interactions on the RMF (root mass fraction) of ten species.
