# Supplementary material for: Effects of Microplastics, Fertilization and Pesticides on Alien and Native Plants
Source: Plants (Basel). 2024 Oct 22;13(21):2947. doi: 10.3390/plants13212947 (PMC11547785; doi:10.3390/plants13212947)

Table S1 Background information of ten species.

Species name	Origin	Life history	Growth form
Coix lacryma-jobi L.	Native	Annual	Graminoid
Cyanthillium cinereum (L.) H. Rob.	Native	Perennial	Forb
Laggera crispata (Vahl) Hepper & J. R. I. Wood	Native	Perennial or annual	Forb
Puhuaea sequax (Wall.) H.Ohashi & K.Ohashi	Native	Perennial	Shrub
Senecio scandens Buch.-Ham. ex D.Don	Native	Perennial	Forb
Ageratina adenophora (Spreng.) R.M.King & H. Rob.	Alien	Perennial	Forb
Bidens pilosa L.	Alien	Annual	Forb
Chromolaena odorata (L.) R.M.King & H.Rob.	Alien	Perennial	Subshrub
Phytolacca americana L.	Alien	Perennial	Forb
Tithonia diversifolia (Hemsl.) A.Gray	Alien	Annual	Forb

Table S2 Details on data transformations in the seven-traits models across the ten species. (sqrt: square root scale; log: logit scale; no: no transformation).

Species	Total	Leaf	Stem	Root	Height	RMF	SLA
Ageratina adenophora	log	log	log	no	log	no	sqrt
Bidens pilosa	no	no	no	log	no	no	log
Chromolaena odorata	sqrt	sqrt	sqrt	log	no	sqrt	log
Coix lacryma-jobi	log	log	log	log	no	no	log
Cyanthillium cinereum	sqrt	log	sqrt	log	sqrt	no	no
Laggera crispata	no	no	no	no	no	no	no
Phytolacca americana	log	no	log	sqrt	no	no	no
Puhuaea sequax	no	log	log	no	no	no	no
Senecio scandens	no	no	no	sqrt	no	no	no
Tithonia diversifolia	no	sqrt	sqrt	no	no	no	log

(Total: total biomass; Leaf: leaf biomass; Stem: stem biomass; Root: root biomass; Height: plant height; RMF: root mass fraction; SLA: specific leaf area)

Figure S1 Effects of nutrients (Nutri) on the total biomass (a), leaf biomass (b), root biomass (c), stem biomass (d) and height (e) of plants. Error bars indicate standard error. Significant effects (asterisk) are indicated in the right corner (details in Table 1).

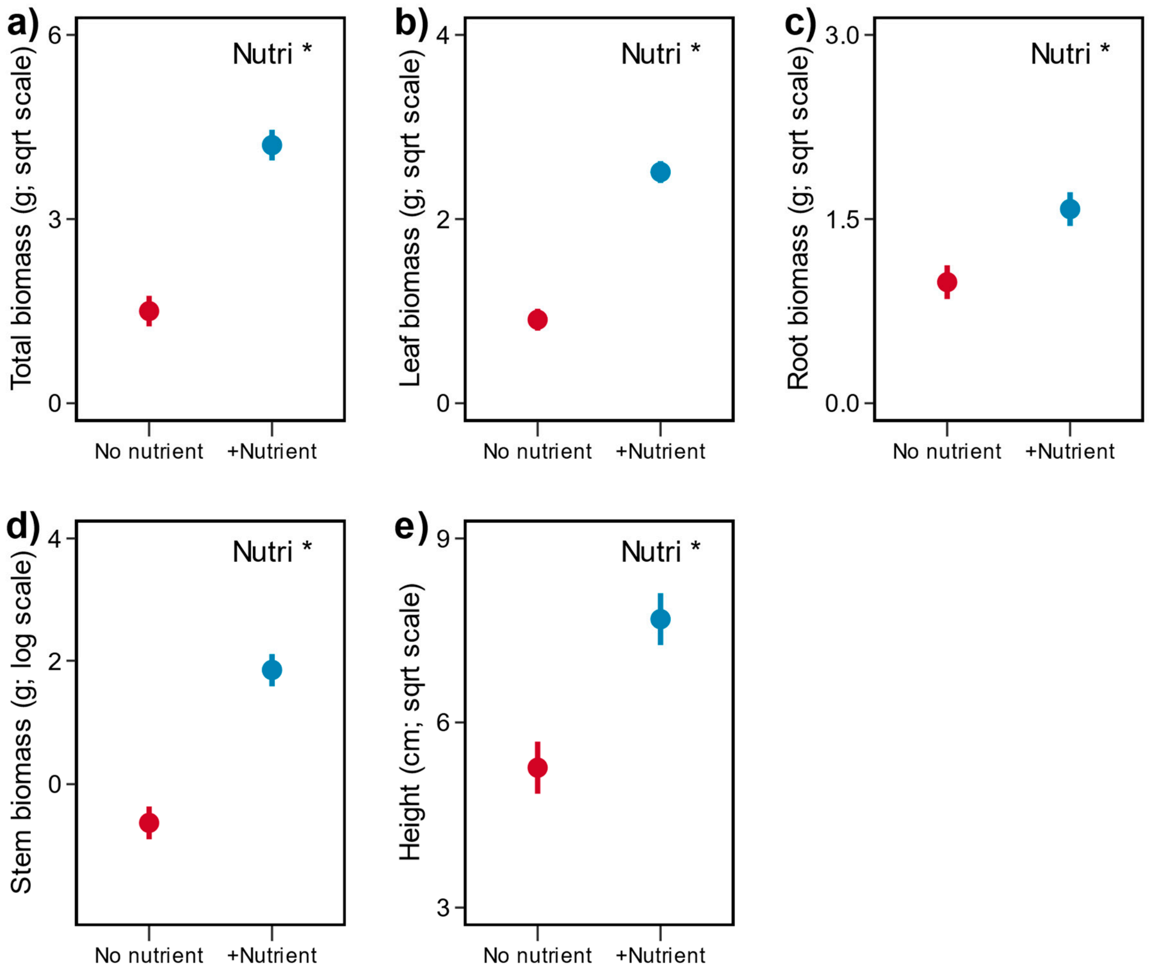

Figure S2 Effects of microplastics, nutrients, pesticides and their interactions on the total biomass of ten species. Significant effects (asterisk) are indicated in the left corner.

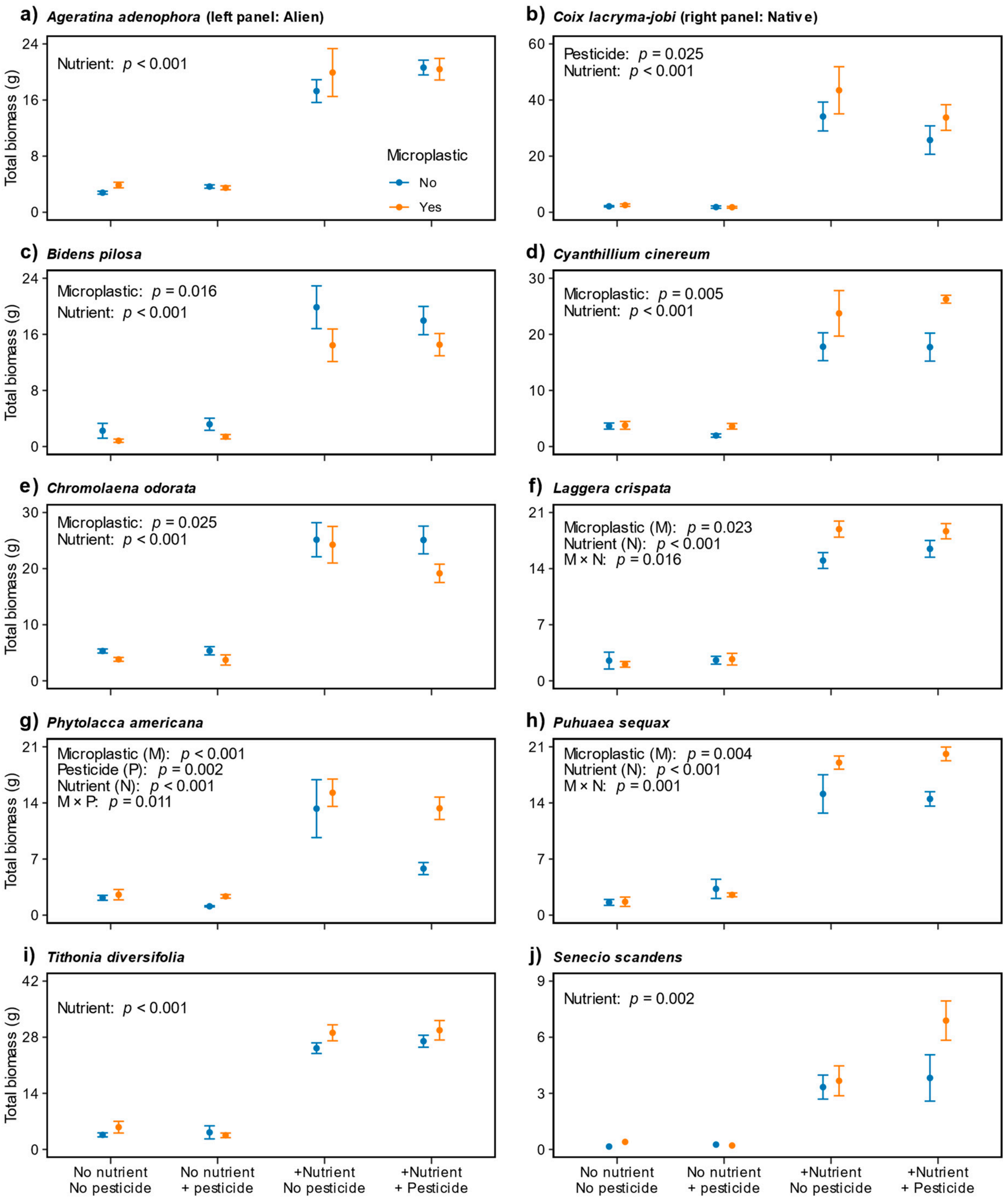

Figure S3 Effects of microplastics, nutrients, pesticides and their interactions on the leaf biomass of ten species. Significant effects (asterisk) are indicated in the left corner.

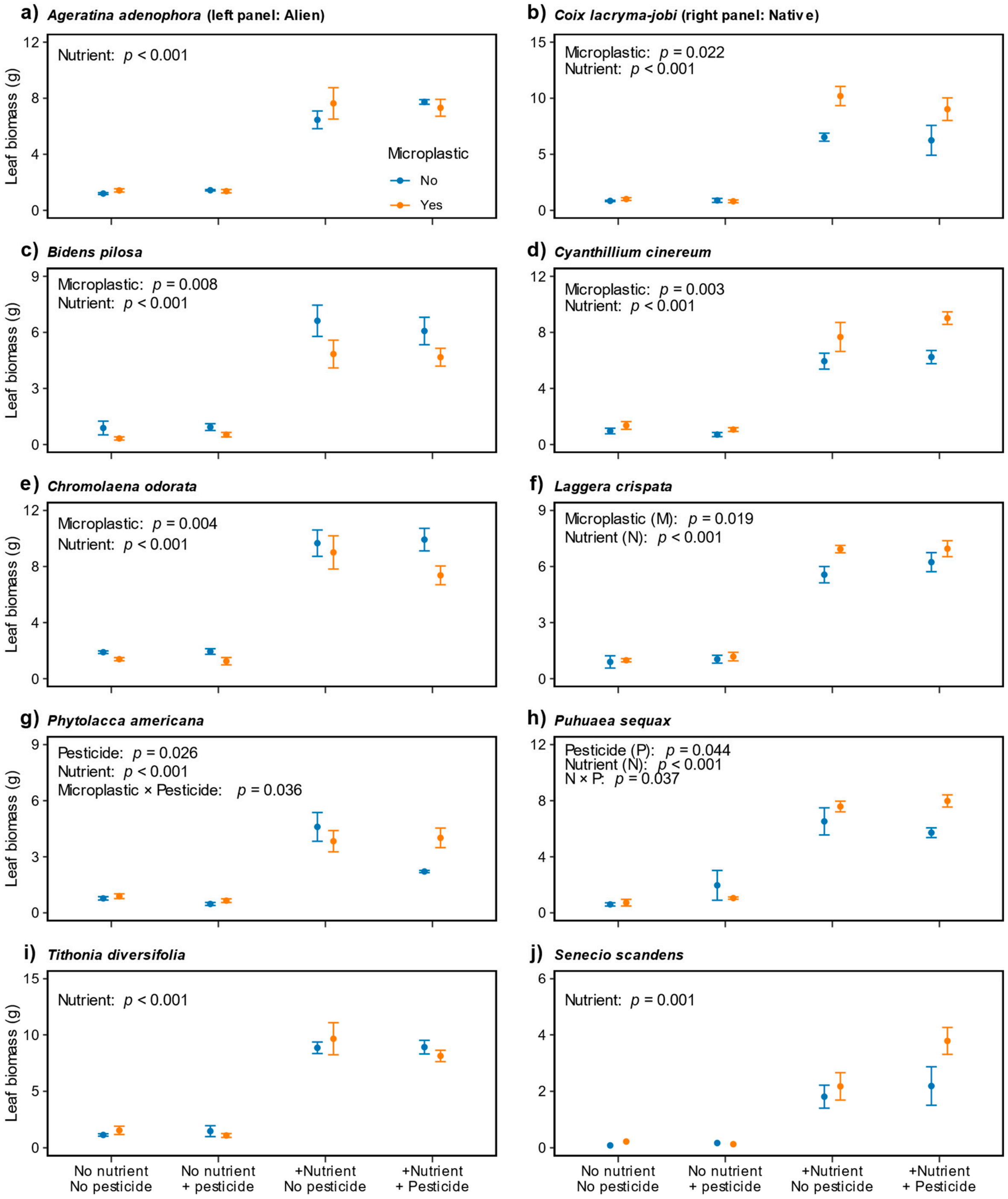

Figure S4 Effects of microplastics, nutrients, pesticides and their interactions on the stem biomass of ten species. Significant effects (asterisk) are indicated in the left corner.

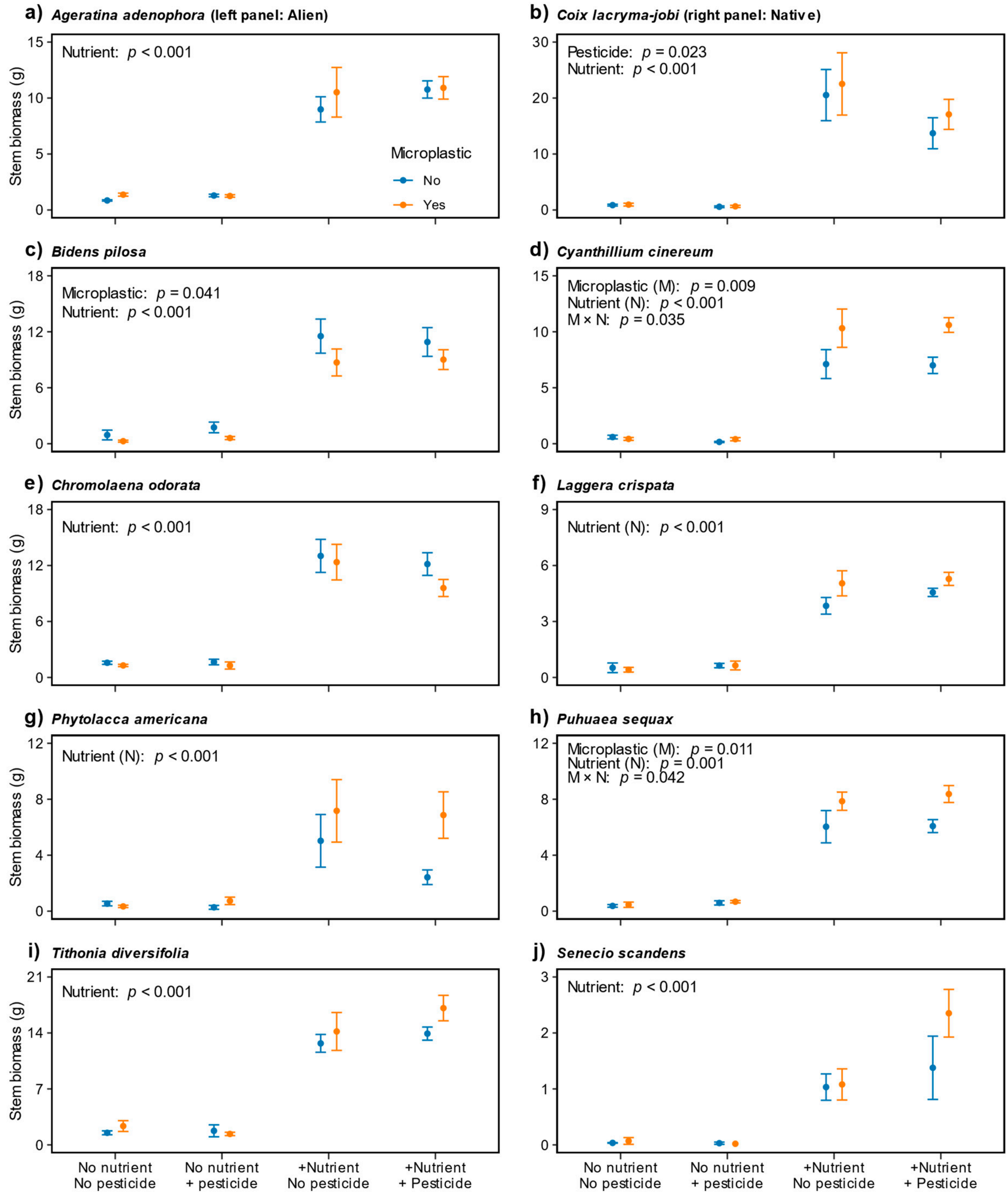

Figure S5 Effects of microplastics, nutrients, pesticides and their interactions on the root biomass of ten species. Significant effects (asterisk) are indicated in the left corner.

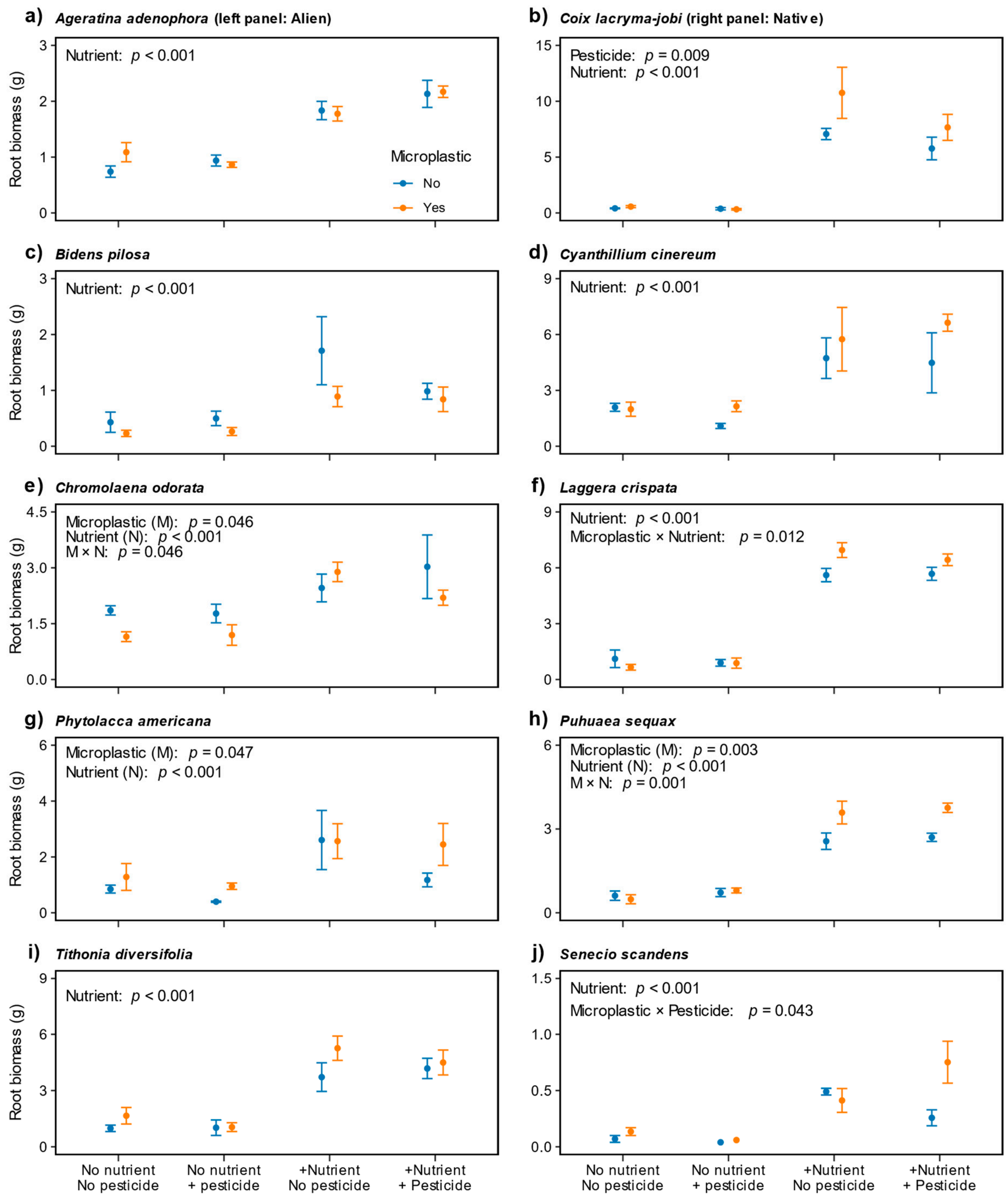

Figure S6 Effects of microplastics, nutrients, pesticides and their interactions on the height of ten species. Significant effects (asterisk) are indicated in the left corner.

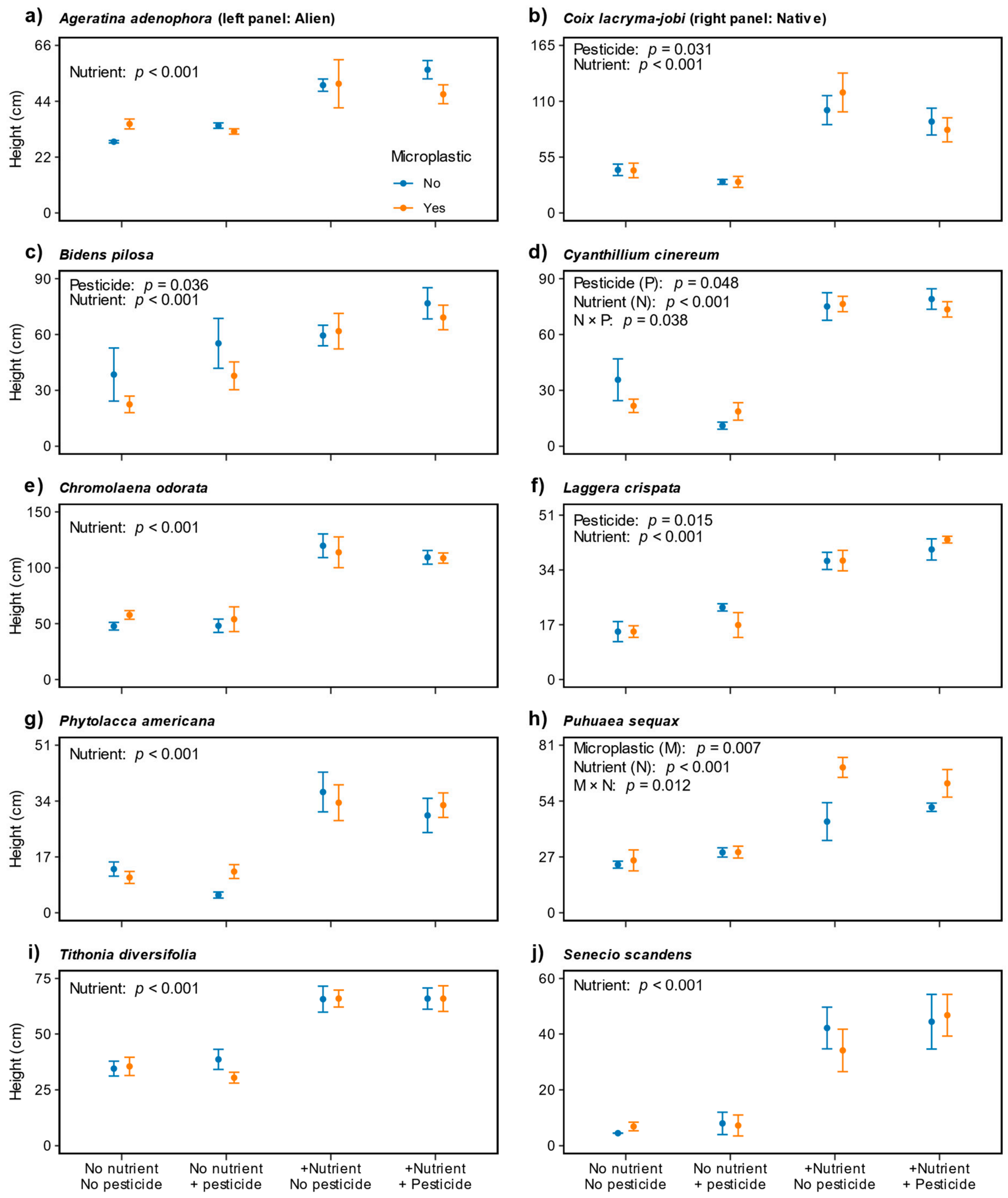

Figure S7 Effects of microplastics, nutrients, pesticides and their interactions on the SLA (specific leaf area) of ten species. Significant effects (asterisk) are indicated in the right corner.

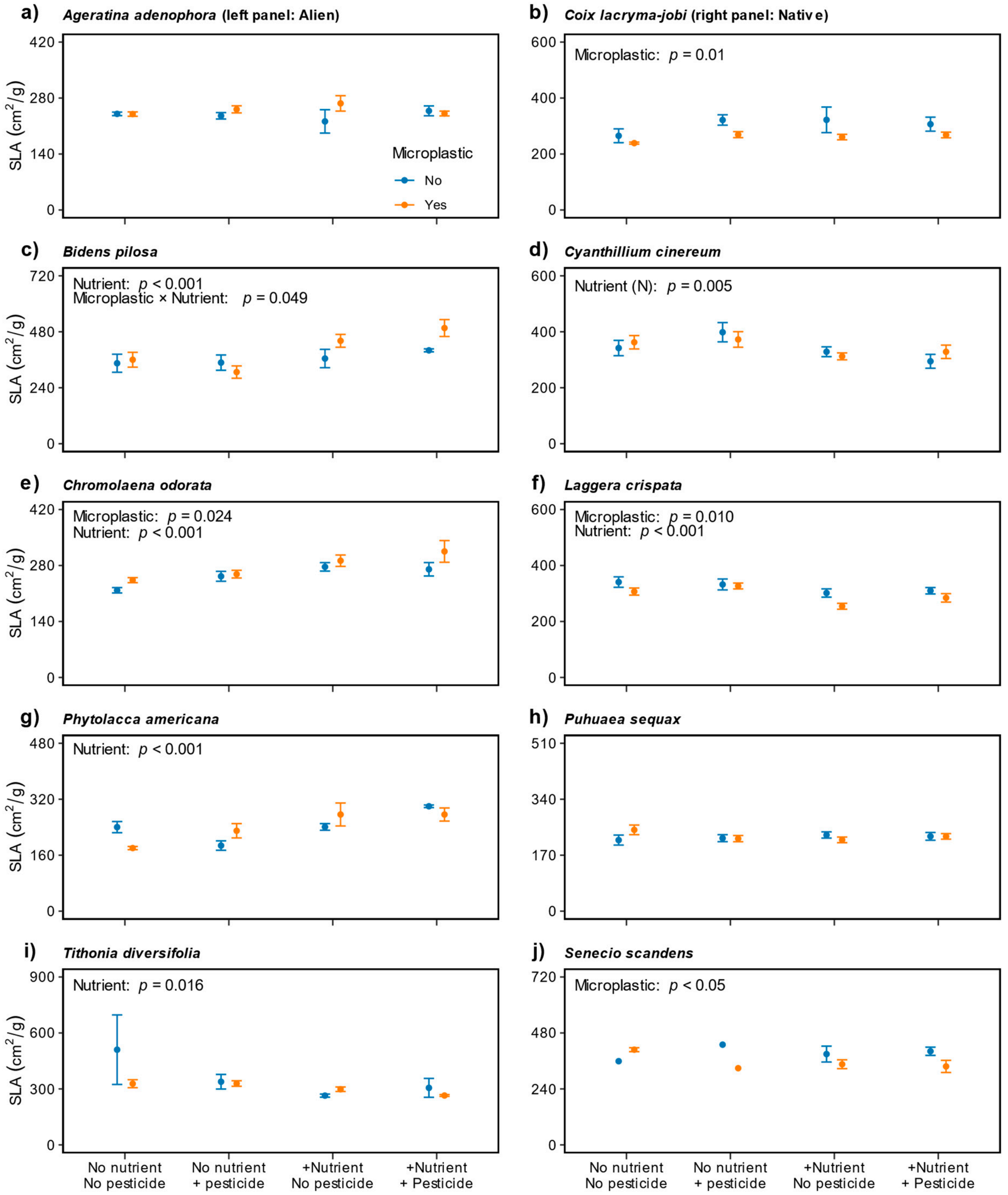

Figure S8 Effects of microplastics, nutrients, pesticides and their interactions on the RMF (root mass fraction) of ten species. Significant effects (asterisk) are indicated in the right corner.

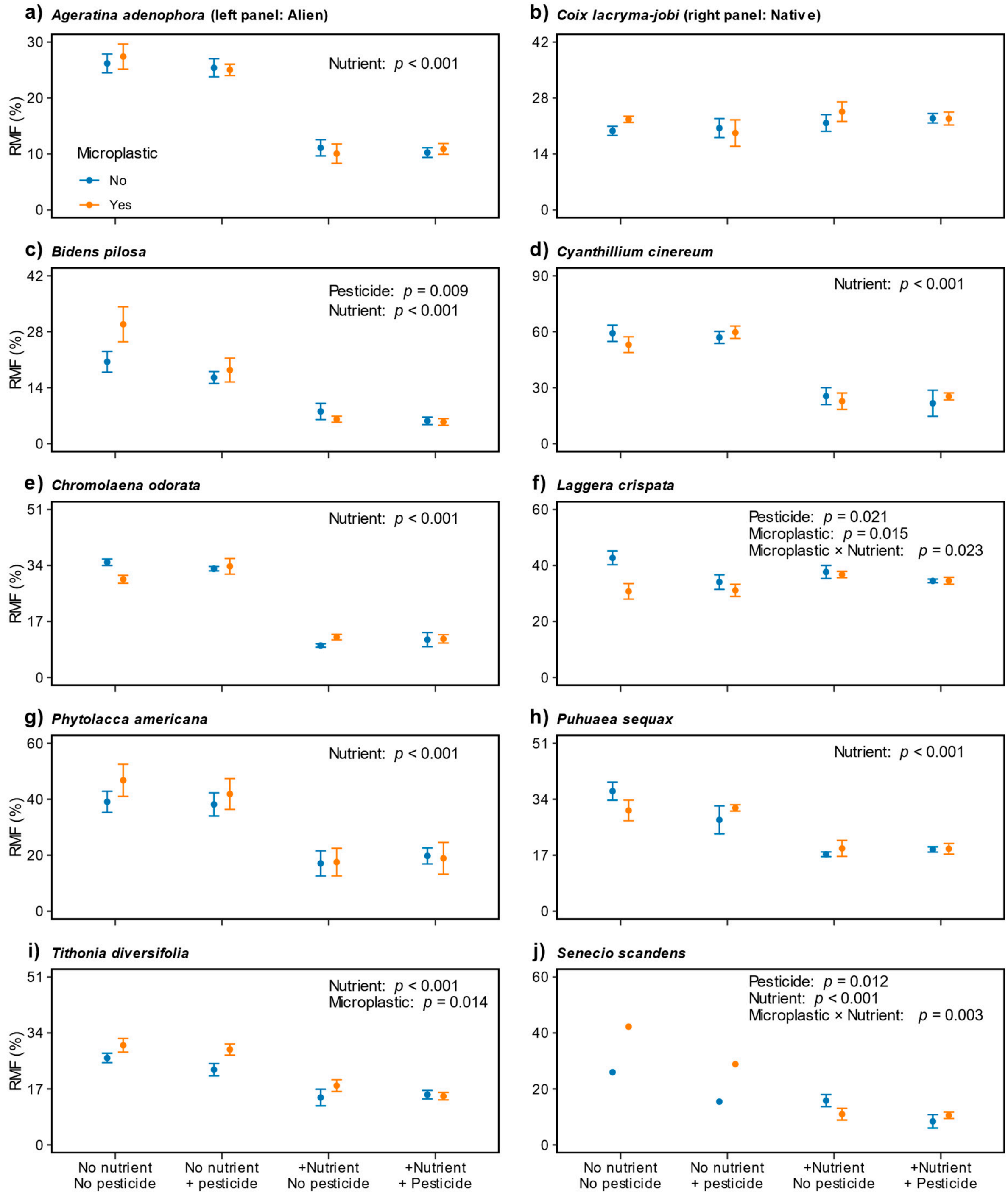

Supplement: Supplementary file 1 [file plants-13-02947-s001.zip › plants-3123553-supplementary.pdf]
